# Vitamin A and Retinoids in Bladder Cancer Chemoprevention and Treatment: A Narrative Review of Current Evidence, Challenges and Future Prospects

**DOI:** 10.3390/ijms22073510

**Published:** 2021-03-29

**Authors:** Larisa Tratnjek, Jera Jeruc, Rok Romih, Daša Zupančič

**Affiliations:** 1 Institute of Cell Biology, Faculty of Medicine, University of Ljubljana, 1000 Ljubljana, Slovenia; larisa.tratnjek@mf.uni-lj.si (L.T.); rok.romih@mf.uni-lj.si (R.R.); 2 Institute of Pathology, Faculty of Medicine, University of Ljubljana, 1000 Ljubljana, Slovenia; jera.jeruc@mf.uni-lj.si

**Keywords:** bladder cancer, vitamin A, retinoids, retinoic acid signalling, chemoprevention, treatment, experimental bladder cancer models, clinical trials, delivery systems, novel targets

## Abstract

Bladder cancer (BC) is the tenth most common cancer worldwide with a high recurrence rate, morbidity and mortality. Therefore, chemoprevention and improved treatment of BC are of paramount importance. Epidemiological studies suggest that adequate vitamin A intake may be associated with reduced BC risk. In addition, retinoids, natural and synthetic derivatives of vitamin A, are intensively studied in cancer research due to their antioxidant properties and their ability to regulate cell growth, differentiation, and apoptosis. Findings from in vivo and in vitro models of BC show great potential for the use of retinoids in the chemoprevention and treatment of BC. However, translation to the clinical practice is limited. In this narrative review we discuss: (i) vitamin A and retinoid metabolism and retinoic acid signalling, (ii) the pathobiology of BC and the need for chemoprevention, (iii) the epidemiological evidence for the role of dietary vitamin A in BC, (iv) mechanistic insights obtained from in vivo and in vitro models, (v) clinical trials of retinoids and the limitations of retinoid use, (vi) novel systems of retinoid delivery, and (vii) components of retinoid signalling pathways as potential novel therapeutic targets.

## 1. Introduction

Vitamin A is a generic term for a group of lipophilic isoprenoids consisting of a cyclic group and a linear chain with a hydrophilic polar group that includes the major biologically active forms retinol, retinal, and retinoic acid (RA) [1]. Since vitamin A cannot be synthesized in the human body, it must be obtained from the diet [2]. The importance of vitamin A for human health was already known to the ancient Egyptians around 1500–1800 B.C., although they did not know vitamin A as such. They recommended compressed animal livers for the treatment of night blindness or nyctalopia. Today we know that the liver is the richest source of vitamin A and that night blindness is caused by vitamin A deficiency (VAD) [3,4,5]. Rhodopsin with its covalently bound cofactor retinal is a major light-sensitive receptor protein involved in visual phototransduction and essential for normal vision. But the importance of vitamin A goes beyond visual health. Vitamin A is a regulator of cell growth and differentiation, embryogenesis, reproduction, epithelial cell integrity, and immune function [1,6,7]. In addition, it has antioxidant properties [8] and plays a role in protecting against oxidative stress damage and inflammation [1,9]. Recent data also indicate that vitamin A regulates the interactions between eukaryotic host cells and symbiotic microbes, as well as the complexity of the microbiome. On the other hand, the microbiome regulates vitamin A metabolism in the host [10,11].

Vitamin A belongs to the retinoids, a group of over 4000 molecules, which are natural and synthetic compounds that are structurally similar or share functional similarities [2,12,13]. Retinoids are classified into four generations based on the time of introduction and structural features: (i) first generation: retinol, retinaldehyde, all-trans RA (ATRA), tretinoin, isotretinoin; (ii) second generation: etretinate, acitretin; (iii) third generation: adapalene, tazarotene, bexarotene; (iv) fourth generation: seletinoid G [14]. The current use of retinoids in medicine is broad, especially in the field of skin health. For example, they are used for the treatment of various inflammatory and keratinization skin diseases (e.g., psoriasis, pityriasis rubra pilaris, lichen planus), as well as basal cell carcinoma [14]. Moreover, retinoids have been used successfully for the treatment of several other cancers, especially acute promyelocytic leukaemia in adults and neuroblastoma in children [15,16].

Bladder cancer (BC), which usually arises from the urothelial cells, is one of the ten most common cancers worldwide. As it has a high recurrence rate of 50–70% and represents a huge social and economic burden [17,18,19], new prevention and treatment strategies are needed. Retinoids are among the best-studied chemopreventive agents for various diseases and are used in clinical practice for chemoprevention and treatment of several cancers [15,20]. Meta-analyses of epidemiological studies indicate that high dietary vitamin A intake reduces the risk of BC [21,22]. Several preclinical studies have shown great potential of retinoids for chemoprevention and treatment of BC, however, translation into clinical use remains limited due to application challenges. Nevertheless, novel synthetic retinoids and retinoid delivery systems have been developed, which, together with the discovery of novel therapeutic targets in the retinoid pathway, offer new opportunities for successful translation of retinoid application into the clinical setting.

## 2. Vitamin A Uptake, Metabolism and Signalling

**Figure 1:**
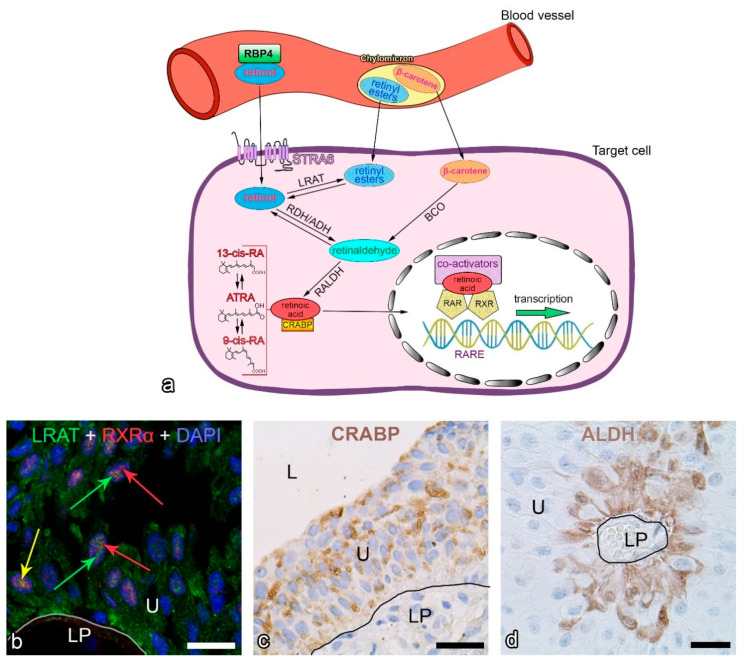
Molecular pathways of vitamin A in the typical human cell (**a**). Immunofluorescence of LRAT and RXRα (**b**) and immunohistochemistry of CRABP (**c**) and ALDH (**d**) in human urothelial cells from biopsy samples of papillary urothelial neoplasms of low malignant potential (PUNLMP). (**b**) LRAT (green) is labelled mainly in the cytoplasm of urothelial cells, while RXRα (red, red arrows) is labelled exclusively in the nuclei of urothelial cells. Some LRAT labelling is detected in the nuclei of urothelial cells (green arrows). The yellow arrow shows co-localization between LRAT and RXRα, which occurs rarely. The nuclei are stained blue with DAPI. (**c**) CRABP (brown) is labelled in the cytoplasm of urothelial cells of all cell layers, whereas (**d**) ALDH (brown) is labelled predominantly in urothelial cells near the basal lamina (black line). Cell nuclei are stained blue with haematoxylin. ADH, alcohol dehydrogenase; ALDH, aldehyde dehydrogenase; ATRA, all-trans retinoic acid; BCO, β-carotene oxygenase; CRABP, cellular retinoic acid-binding protein; DAPI, 4′,6-diamidino-2′-phenylindole dihydrochloride; L, lumen of bladder; LP, lamina propria; LRAT, lecithin retinol acyltransferase; RA, retinoic acid; RALDH, retinal dehydrogenase; RAR, retinoic acid receptor; RARE, RA response element; RBP4, retinol-binding protein 4; RDH, retinol dehydrogenase; RXR, retinoid X receptor; STRA6, stimulated by retinoic acid 6; U, urothelium; white or black line, basal lamina. Scale bars: 20 µm (**b**), 50 µm (**c**,**d**).

Dietary vitamin A occurs in two main forms: preformed vitamin A (retinol, retinal, RA, retinyl esters), found mainly in animal food sources (meats, especially liver, fish and dairy products), and provitamin A carotenoids with the potential to form vitamin A (such as α-carotene, β-carotene, β-cryptoxanthin), found in plants [3,23]. The main source of retinoids for the human body is β-carotene, which is metabolized in the intestine to retinal and retinol and subsequently transported through the blood by retinol-binding protein 4 (RBP4) (Figure 1a). Retinoids can also be absorbed directly from the food (retinyl esters and β-carotene), packaged into chylomicrons and transported into the general blood circulation via the lymphatic system [16] (Figure 1a). The majority of dietary retinoids are delivered and stored in the liver [12]. When needed, retinoids are transported from the liver throughout the body via the bloodstream in the form of retinol bound to RBP4. Target cells express the transmembrane-spanning receptor stimulated by retinoic acid 6 (STRA6), which mediates cellular uptake of retinol [24,25] (Figure 1a). Cells can also take up retinyl esters and β-carotene from chylomicrons via lipoprotein-specific receptors [26].

Once inside the cell, retinoids, which are poorly soluble in aqueous solutions, bind to cellular retinoid-binding proteins (cellular retinol-binding protein-CRBP, cellular retinoic acid-binding protein-CRABP, fatty acid-binding protein 5–FABP5) to be effectively transported, metabolized and functionalized [27]. Retinol and retinyl esters are not biologically active and are activated via a series of oxidation steps. Retinol is converted to retinaldehyde by retinol dehydrogenases (RDHs) or alcohol dehydrogenases (ADHs). Another source of retinaldehyde comes from β-carotene through conversion mediated by β-carotene oxygenase (BCO) (Figure 1a). Retinaldehyde is oxidized by retinal dehydrogenases (RALDHs), also known as aldehyde dehydrogenases (ALDHs), to RA, the most active metabolite [16,28] (Figure 1a,d). The esterification of retinol to retinyl esters is mediated by lecithin retinol acyltransferase (LRAT) [25] (Figure 1a,b).

RA has several isomeric forms: ATRA, 9-cis-RA, 11-cis-RA, 13-cis-RA etc. [29]. RA can bind to CRABP and migrate to the nucleus for receptor binding (Figure 1a,c) or is transformed to oxidized and mostly inactive compounds by the enzyme cytochrome P450 (CYP26) [30]. In the nucleus, RA regulates gene expression by binding to retinoic acid receptors (RARs), retinoid X receptors (RXRs) (Figure 1a,b) or to other proteins such as peroxisome proliferator-activated receptor (PPAR), which in turn activate transcription of their downstream target genes [16,28]. The nuclear receptors RAR and RXR consist of three receptor types α, β and γ [31]. RA exerts multiple effects by binding to its receptors in the form of dimers, which in turn bind to the corresponding RA response elements (RARE) located in the regulatory regions of target genes [13] (Figure 1a). Different RA isomers activate different receptors, leading to different biological effects. Indeed, more than 500 genes have been identified responding to RA signalling [32].

## 3. Pathobiology of Bladder Cancer and the Chemoprevention Need

BC is the tenth most common cancer worldwide with nearly 600,000 new cases in 2020, accounting for approximately 3% of all cancer diagnoses [17,19,33]. More than 90% of all BC cases, especially in developed countries, are urothelial (transitional cell) carcinomas, while primary squamous cell carcinomas, adenocarcinomas, small cell carcinomas, and other tumours are less common [34]. Urothelial carcinoma is highly associated with chemical exposure, such as occupational exposure to carcinogenic aromatic amines and tobacco smoke. Squamous cell carcinoma, which accounts for approximately 5% of BC worldwide, is more common in Africa due to schistosomiasis, a protozoan infection that promotes inflammation [35].

**Figure 2:**
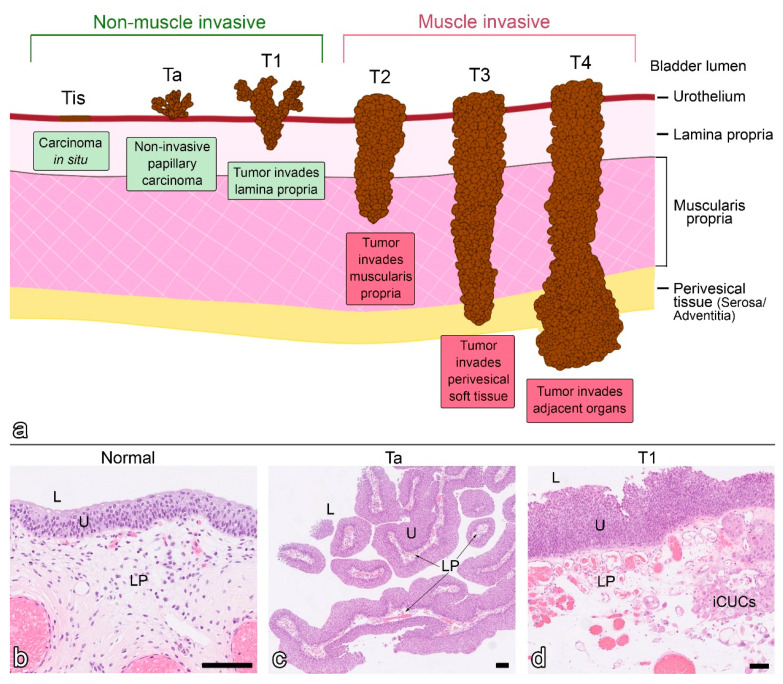
Staging of bladder cancer (BC) according to the Tumour, Node, Metastasis classification system (**a**). Tumours that are confined to the mucosa, including carcinoma in situ (Tis) and papillary tumours (Ta), or that have not invaded beyond the lamina propria (T1) are grouped under the term non-muscle invasive BC, whereas those that infiltrate the muscularis propria and beyond (T2–T4) are grouped under the term muscle invasive BC. Representative histological sections of human normal bladder mucosa (**b**), non-muscle invasive papillary BC (Ta) (**c**) and papillary BC infiltrating the lamina propria (T1) (**d**). iCUCs, invading cancer urothelial cells; L, lumen of bladder; LP, lamina propria; U, urothelium. Scale bar, 100 µm (**b**–**d**).

The urothelium is the innermost layer of the urinary bladder (Figure 2), which consists of basal, intermediate, and superficial urothelial cells. Beneath the urothelium, which maintains the tight blood-urine barrier, are a basement membrane and a lamina propria. The lamina propria consists of a zone of loose connective tissue with delicate vessels and thin bundles of muscularis mucosae and a zone of connective tissue with larger vessels. Below, there is the muscularis propria or detrusor, surrounded by the serosa, a thin layer of connective tissue covering the bladder dome and continuing to the peritoneal layer of the abdominal wall. In the areas of the bladder where serosa is not present, the outer layer is the adventitia [36,37] (Figure 2). According to the TNM (Tumour, Node, Metastasis) classification system, the term perivesical tissue is used for serosa and adventitia.

BC is a heterogeneous disease with a spectrum of pathologies and clinical outcomes [38]. Based on the depth of invasion through the bladder wall, BCs can be classified into those confined to the urothelium, i.e., non-invasive and invasive. Non-invasive BC is subdivided into high- and low-grade papillary tumours (Ta according to the TNM classification system) and urothelial carcinoma in situ (CIS–stage Tis), which is defined as a high-grade, flat, non-invasive lesion confined to the mucosa. Invasive BC can be further subdivided into the tumours invading the lamina propria and muscularis propria (T1 and T2, respectively) or extending beyond the bladder wall into the perivesical adipose tissue and sometimes into the adjacent organs (T3 and T4, respectively) [39] (Figure 2). For therapeutic purposes, Tis, Ta and T1 tumours are grouped under the term non-muscle invasive BC (NMIBC) and T2–T4 tumours are grouped under the term muscle invasive BC (MIBC) (Figure 2). NMIBC is present in approximately 75% of patients with an initial diagnosis of BC [40]. NMIBC is usually treated by transurethral resection of the bladder (TURB). Sometimes TURB is followed by intravesical instillation of chemotherapeutic agents (mitomycin C) or Bacillus Calmette Guerin (BCG) [41]. NMIBC is characterized by an extremely high recurrence rate (50–70%), requiring systematic follow-up of patients decades after initial treatment [42]. Nevertheless, NMIBC has a low propensity for progression (10-15%) and a 5-year survival rate of 90% [43]. The current standard treatment for MIBC is radical cystectomy, but still, a high rate of metastasis and a 5-year survival rate of <50% are observed [43,44]. Furthermore, BC shows a steady increase in its incidence and prevalence and is associated with high morbidity. The number of BC cases and deaths is expected to increase in the future due to the estimated population growth and aging, as most BC are diagnosed at the age of >65 years. Therefore, the development of effective chemoprevention is of utmost importance.

The key players in the initiation and recurrence of BC are urothelial cancer stem cells (CSCs), yet their identification remains elusive. Several studies suggested that keratin 5, keratin 14, sonic hedgehog (Shh) and β-catenin positive basal urothelial cells may be the origin of all urothelial cancers [45,46,47]. This hypothesis has been challenged by the suggestion that non-invasive papillary carcinomas arise from intermediate urothelial cells, whereas MIBC arise from the transformation of keratin 5 negative basal cells [45].

Signalling pathways are another critical point in BC development and progression. It is well known that the retinoid signalling is often dysregulated in cancer [48]. It was reported that LRAT expression is significantly reduced in human BC and an inverse correlation has been shown between LRAT expression and increasing tumour stage [49]. Furthermore, CRBP1 was demonstrated to be downregulated in BC through the CpG hypermethylation of the promoter region [50]. Additionally, the expression of retinoic acid-related orphan receptor C (RORC), which functions as a DNA-binding transcription factor, was downregulated in tumour tissues from BC patients [51]. Lower RORC expression was found in advanced tumours and those resistant to chemotherapy [51]. In contrast, ALDH1A1 and its putative downstream target TUBB3 were overexpressed in BC, and a clinical survival database revealed that TUBB3 expression may be associated with poor prognosis in BC patients [52]. Therefore, it is clear that the retinoid signalling pathway is altered during bladder carcinogenesis.

## 4. The Role of Dietary Vitamin A in Bladder Cancer: The Epidemiologic Evidence

Vitamin A and retinoids are among the best-studied micronutrients and have great potential for prevention and cancer treatment due to their differentiating, antiproliferative, pro-apoptotic, and antioxidant effects combined with selectivity, high receptor binding affinity, and ability to directly modulate gene expression programs [15,53].

An association between VAD and the incidence of cancer was first demonstrated around 1920 in animal studies showing that VAD increased the incidence of spontaneous and carcinogen-induced tumours [54,55,56,57]. In 1979, a retrospective study of human dietary habits and BC showed an increased risk in people with low vitamin A intake [58], implicating vitamin A as a potential agent for BC prevention. Despite the fact that vitamin A is present in a wide variety of foods, many people do not consume this nutrient adequately due to malnutrition or selective diets, leading to VAD. Therefore, the impact of vitamin A intake on BC risk has important public health implications [21,59,60].

Typically, VAD develops in environments of ecological, social and economic deprivation. Recent analysis showed a decline in VAD prevalence primarily due to decrease in East and Southeast Asia, Oceania, Latin America and the Caribbean, while it remains high in South Asia and sub-Saharan Africa [61]. Moreover, we have to point out that Western diets containing mainly processed foods can lead to subclinical VAD, which often goes unnoticed but may be implicated in the development of some cancers [62].

The highest rates of BC are observed in developed countries in Europe, Northern America, and Western Asia, but also in Syrian, Israeli, Egyptian and Turkish men. Approximately threefold lower rates are seen in Southeast Asia (except Japan) and in Latin America and Northern Africa in both sexes, and the lowest in Sub-Saharan Africa and some Middle Eastern and Central Asian countries [17,19,63,64].

Looking at the global distribution of BC incidence and VAD, the association between the two is not immediately apparent. Nevertheless, numerous population-based epidemiological studies investigated the relationship between dietary vitamin A and BC risk, including several meta-analyses [21,22,65,66]. While older studies concluded that dietary retinol and β-carotene play a minimal role in BC [65], more recent studies show a preventive effect of vitamin A on BC. A meta-analysis of 25 studies investigating the quantitative effects of vitamin A on BC revealed that high vitamin A intake and high blood retinol levels were associated with a reduced risk of BC [21]. The most recent meta-analysis of 22 studies conducted in Northern America, Europe, or Japan (19 of which were included in the previous analysis by Tang et al. [21]) indicated that the risk of BC decreased by 76% for every 1 µmol/L increase in circulating concentrations of α-carotene, and by 27% for every 1 µmol/L increase in circulating concentrations of β-carotene. When comparing high and low total dietary carotenoid intake, high intake was associated with a 15% reduced risk of BC in men [22].

On the other hand, very high intakes of preformed vitamin A present in animal foods and pharmaceutical supplements can cause acute or chronic toxicity, while very high doses of provitamin A (carotenoids) from plants do not. Acute hypervitaminosis A is a consequence of the ingestion (usually accidental) of more than 300,000 IU of vitamin A as a single dose or several repeated doses over a few days, whereas chronic hypervitaminosis A is a result of continued ingestion of more than 100,000 IU daily for months or years [67]. In addition, a single dose of more than 25,000 IU of vitamin A may be teratogenic if consumed between the 15th and 60th day after conception [68].

Although the evidence for the correlation between BC aetiology and diet are not yet conclusive, diet is considered one of the modifiable risk factors for BC prevention [69,70]. There is still a large gap to be filled in understanding the molecular mechanisms by which vitamin A affects urothelium and urothelial carcinogenesis. To address this issue, various in vivo and in vitro models mimicking human BC have been widely used.

## 5. Experimental Models of Bladder Cancer Play a Key Role in Understanding the Chemopreventive and Therapeutic Effects of Vitamin A and Retinoids

Several retinoids, such as ATRA, 13-cis-RA, and N-(4-hydroxyphenyl)-retinamide (4-HPR, or fenretinide), showed promising chemopreventive effects on BC both in vitro and in vivo (Table 1 and Table 2, respectively). In vitro studies suggest that retinoids exert their chemopreventive effects on BC through cytostatic, pro-apoptotic, growth inhibitory, cell cycle distribution, and gene expression modulating/regulating functions [71,72,73,74,75,76]. The study by Boström et al. suggested that retinoids may downregulate the expression of matrix metalloproteinases (MMPs), which play an important role in the process of degradation of extracellular matrix essential for tumour growth and invasion [77]. The mechanism of retinoid BC chemoprevention may also include reversion of epithelial-mesenchymal transition, a key process in cancer cell invasion and migration. Wang et al. showed that the synthetic retinoid 4-HPR increased the expression of E-cadherin in invasive BC cell lines and induced the translocation of β-catenin from the nucleus to the cytoplasm, resulting in an altered BC cell morphology that resembles epithelial rather than invasive cancer cells, presumably leading to reduced cell infiltration [78].

**Figure 3:**
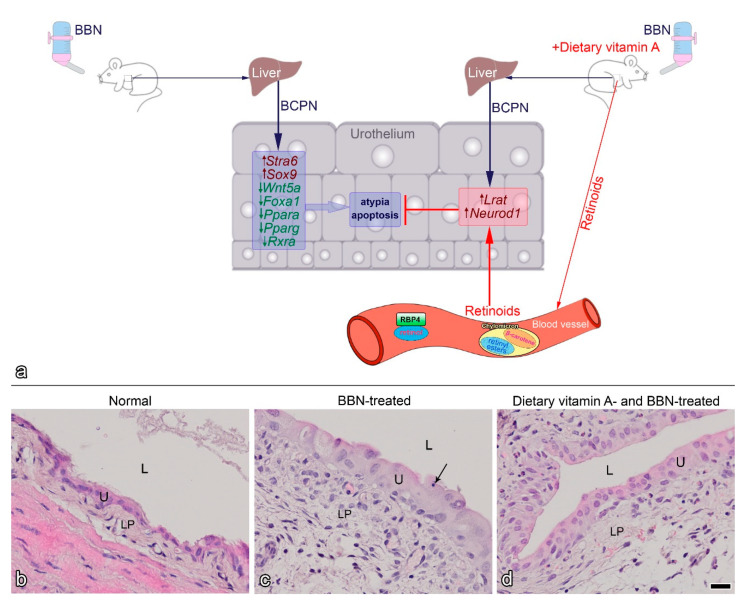
Proposed model of BBN-induced early bladder carcinogenesis and the effects of dietary vitamin A (modified from [89]) (**a**). BBN (N-butyl-N-(4-hydroxybutyl)-nitrosamine) administered orally to experimental mice via drinking water is metabolized in the liver to BCPN (N-butyl-N-(3-carboxypropyl)-nitrosamine), which reaches urothelium through urine and can affect urothelial cells. BCPN binds to cellular macromolecules, including DNA, and initiates the early carcinogenic process in urothelial cells, manifested by altered RA signalling. Expression of the RA receptor *Stra6* and the transcription factor *Sox9* is upregulated, while the expression of the RA pathway regulator *Wnt5a* and the transcription factors *Foxa1*, *Ppara*, *Pparg* and *Rxra* is downregulated. Histologically, early urothelial carcinogenesis reveals as urothelial atypia and increased apoptosis. When experimental mice received BBN and dietary vitamin A in the form of a vitamin A-rich diet, retinoids reach the urothelium via the blood circulation. RA signalling is altered in urothelial cells in a different way, compared to BBN-treatment alone. In this case, the expression of *Lrat* and the transcription factor *Neurod1* are upregulated, while the expression of other genes is not significantly altered. Retinoids from the blood reduce BBN-induced atypia and apoptosis. Representative histological section of an early mouse bladder carcinogenesis model: (**b**) normal bladder mucosa. (**c**) two weeks of BBN treatment causes early carcinogenesis with characteristic atypia and apoptotic bodies (black arrow). (**d**) three weeks of dietary vitamin A together with 2 weeks of BBN treatment decrease urothelial changes. L, lumen of bladder; LP, lamina propria; U, urothelium. Scale bar: 20 µm (**b**–**d**).

Animal studies have examined chemopreventive and therapeutic effects of retinoids in carcinogen-based in vivo models of BC (Table 2). In these models, tumours develop after animals are treated with carcinogens that mimic environmental exposures known to be a major cause of BC. Carcinogen-based BC models recapitulate the high mutational burden and complexity of human BC [79,80]. Among the carcinogens, the most prevalently used is N-butyl-N-(4-hydroxybutyl)-nitrosamine (BBN), a compound closely related to some of the carcinogens found in tobacco smoke, showing remarkable specificity for the urinary bladder [81,82]. Animals orally administered BBN (Figure 3a) develop bladder tumours recapitulating the histology of human BC and its morphological, biological, and molecular features [83]. To a lesser extent, the chemopreventive effects of retinoids in BC have also been investigated in N-methyl-N-nitrosourea (MNU) and N-4-(5-nitro-2-furyl)-2-thiazolylformamide (FANFT) in vivo models of BC (Table 2). MNU is a direct-acting carcinogen that is locally instilled into the bladder, and MNU-induced BC in animals displays an immunophenotype similar to human urothelial carcinoma [84]. FANFT is a heterocyclic nitro compound and an indirect chemical carcinogen that stimulates the bladder mucosa to develop carcinoma when animals are fed with FANFT [80].

The study of early bladder carcinogenesis using the BBN model showed that dietary vitamin A (supplemented as retinyl acetate) decreased BBN-induced urothelial atypia and apoptosis [89] (Figure 3). Moreover, during early bladder carcinogenesis RA signalling was altered as the expression of several genes was up- or downregulated, while a vitamin A-rich diet prevented this altered expression. In fact, dietary vitamin A together with BBN treatment resulted in upregulation of *Lrat* and the transcription factor *Neurod1* (Figure 3a). In addition, LRAT was observed to be translocated from the cytoplasm to the nuclei of urothelial cells in BBN-treated animals [89]. These results suggest that dietary vitamin A indeed alters cancer-related dysregulation of retinoid signalling and gene expression at early stages of cancer transformation.

It is important to emphasize that anti-cancer activity varies between different retinoid derivatives. Synthetic retinoids (4-HPR and CD437–also known as Ro 472077) have been shown to have stronger effects on growth inhibition and apoptosis than naturally occurring retinoids, e.g., ATRA [74]. Different retinoids induced the expression of different nuclear retinoid receptors (*RARα*, *RARβ*, *RARγ*) and differentially altered the expression of apoptosis-associated genes (*p53*, *GADD45*, *bcl-2*, *casp3*) [74,88]. Moreover, it was shown that ATRA treatment was not always effective due to some resistance mechanisms and that ATRA could even induce a dose- and time-dependent cell proliferation [86]. A similar effect was also shown for bexarotene (also known as LGD1069 or Ro 26-445, brand name Targretin), which increased the incidence and size of tumours that developed in the BBN model [90]. On the other hand, the combination of retinoids, e.g., 4-HPR, with the chemotherapeutic agents, such as adriamycin (ADM), increased the antitumour effects of the chemotherapeutic agents compared to the antitumour effects when both chemicals were used separately [91].

Another important effect of retinoids demonstrated in in vivo models is the discovery that delayed administration of retinoids also inhibits urinary bladder carcinogenesis [97,101]. For example, delaying the administration of a 13-cis-RA supplement for several weeks after the last administration of BBN to rats did not result in a loss of the chemopreventive effect of 13-cis-RA [97]. This is important for the clinical settings because the onset of retinoid administration in a clinical situation would also likely be delayed with respect to the earliest preneoplastic changes in BC patients.

Unfortunately, the preclinical studies have shown limited predictive potential for the clinical trials. All animal studies with retinoids utilized carcinogen-based BC models, whereas retinoid effects have not been studied in engraftment models, in which cells or tissues are grown in recipient hosts, or in genetically engineered mouse models based on activation or inactivation of gene function in the bladder. Therefore, we believe that combining different in vivo models in chemopreventive studies with retinoids could be the way to improve the predictive potential and translate preclinical experiments into clinical trials with positive outcome. We must also point out that interspecies variations must be considered for a correct interpretation of the results. For example, Chopra et al. highlighted the differences in the expression and distribution of PPAR and RXR isoforms between rat and human urothelium, which may underlie a different response to PPAR agonists [106]. This interspecies gap can be overcome by ex vivo studies on human biopsy specimens, which are extremely under-researched. Moreover, carefully designed clinical trials utilizing promising retinoids and retinoid/chemotherapeutic combinations are of utmost importance.

## 6. Clinical Trials of Retinoids for Chemoprevention and Treatment of Bladder Cancer and Limitations of Their Use

Retinoids have been successfully used in several clinical applications, such as the treatment of acute promyelocytic leukaemia with orally administered ATRA [107] and high-risk neuroblastoma with 13-cis-RA [108]. Clinical trials of BC chemoprevention with retinoids (Table 3) have often failed, have not shown efficacy, and have not produced results comparable to in vivo and in vitro studies. It is important to stress out that there have been only a handful of clinical trials conducted, some with small group sizes and some without a placebo group that consequently may not have resulted in statistical significance (Table 3).

A phase III multicentre randomized study in patients with Ta tumours treated with BCG found no benefit from the synthetic retinoid 4-HPR [113,120]. Nevertheless, a subgroup analysis showed that high-risk patients co-treated with 4-HPR and BCG had a lower risk of recurrence compared to the placebo group. Additionally, 4-HPR was shown to decrease plasma levels of insulin-like growth factor I (IGF) in patients with superficial BC [112]. Given the increasingly recognized importance of circulating IGFs in the pathogenesis of various solid tumours, these findings strengthen the rationale for further investigation of 4-HPR as a chemopreventive agent for BC.

The pharmacological use of retinoids encounters several limitations, such as the low concentrations of retinoids at the tumour site, short half-life, poor water solubility, susceptibility to light, heat, and oxidants, and rapid degradation during digestion resulting in low bioavailability and bioaccessibility [20,86]. One of the ways to increase retinoid plasma levels is to combine retinoid treatment with agents that inhibit retinoid degradation, which was tested in a BC clinical trial. The study of Ta and T1 BC patients showed that treatment with a combination of ATRA and ketoconazole (a potent inhibitor of RA-catabolizing cytochrome P450s) significantly improved patient survival and reduced the recurrence rate compared with the control group [118].

One of the limitations to the successful use of retinoids for chemoprevention and treatment is also retinoid resistance. Many potential mechanisms have been proposed for retinoid resistance, including reduced retinoid uptake, increased ATRA catabolism by P450s (CYP26), active drug efflux by membrane transporters, downregulated expression of various RAR genes (promoter methylation), altered expression of co-activators or downstream target genes, and changes in the activities of other signalling pathways [15]. Lu et al. demonstrated a positive correlation between the expression of octamer-binding transcription factor (Oct4) and tumour recurrence in BC. Furthermore, inhibition of Oct4 by ATRA synergistically increased sensitivity to the chemotherapeutic agent cisplatin in preclinical BC studies [121]. Therefore, inhibition of Oct4 could be a therapeutic strategy to overcome drug resistance and reduce the recurrence rate. Combining retinoids with epigenetic drugs also shows great potential to restore tumour response to retinoids. For example, histone acetylation regulates gene transcription so it was proposed that it could restore tumour sensitivity to retinoids. Indeed, the combination of 13-cis-RA and the histone deacetylase (HDAC) inhibitor entinostat was shown to induce histone acetylation in patients with solid tumours including urothelial carcinoma. Although no tumour responses were observed, further evaluation of this combination is warranted [119].

Finally, the use of retinoids in the clinical practice is also limited because long-term administration of natural retinoids is associated with toxicity manifested by hepatic and lipid changes, dry skin, teratogenicity, and bone and connective tissue damage [122]. Treatment with the synthetic retinoid etretinate has been shown to significantly reduce the annual transurethral resection rate in patients with superficial papillary bladder tumours. However, significant cardiac toxicity occurred in the etretinate group [114,115]. To reduce the toxicity of retinoids, novel synthetic retinoids are being developed. For example, the newly developed synthetic retinoid WYC-209 inhibited the growth of tumour repopulating cells of several cancer cell lines (human melanoma, lung cancer, ovarian cancer, and breast cancer) and inhibited lung metastasis in vivo, with low in vivo toxicity [123].

## 7. Novel Retinoid Delivery Systems

One approach to avoid retinoid degradation, increase bioavailability and bioaccessibility, and reduce toxicity is to encapsulate retinoids in various drug delivery systems such as nanoparticles, micelles, liposomes, or bind them to nanoparticles, proteins or polymers [20]. For example, conjugation of RA to nanoparticles such as RA-poly(ethylene glycol)-thiol gold nanoparticle conjugates showed superior activity against the cervical carcinoma cell line compared to free RA, which is attributed to increased rates of drug transport through nanoparticle uptake compared to passive diffusion of free drug [124]. It has been demonstrated that a nanoformulation of 4-HPR complexed with a solubilizing excipient 2-hydroxypropyl-beta-cyclodextrin (nanofenretinide) was shown to increase the bioavailability and therapeutic efficacy of 4-HPR in vitro and in vivo in the absence of macroscopic toxic effects [125]. Next, a 20% soy oil-in-water emulsion of 4-HPR was developed and a phase I study in patients with malignant solid tumours demonstrated a manageable safety profile and achieved higher plasma steady-state concentrations of the active metabolite compared to previous formulations [126].

Novel retinoid-based formulations also show great potential against CSCs. For example, a nano-micellar formulation of 4-HPR based on its encapsulation in the lipid matrix displayed pronounced antitumour activity against lung, colon, and melanoma CSCs both in vitro and in vivo, in the absence of systemic toxicity, suggesting its potential usefulness for the treatment of solid tumours of various origins [127]. Moreover, ATRA and the chemotherapeutic agent doxorubicin were simultaneously encapsulated in the same nanoparticle, which improved the suppression of breast tumour growth while synergistically reducing the incidence of CSCs in preclinical settings [128].

The anticancer efficiency of encapsulated retinoids can be further enhanced when combined with immunotherapy. For example, the lipid-coated biodegradable hollow mesoporous silica nanoparticles with co-encapsulation of ATRA, doxorubicin and interleukin-2 (IL-2) showed great potential for developing a viable strategy to remodel the tumour immune microenvironment and achieve enhanced antitumour effect [129].

Finally, nanoencapsulation may enhance the effect of dietary vitamin A supplementation. Novel carotenoid delivery systems have gained much attention in the food industry due to their enhanced absorption and bioavailability. After oral ingestion, nanocarriers can easily penetrate the mucus barriers, resulting in better cellular uptake [130,131,132]. Currently, polymeric nanocapsules are the most widely used due to their high encapsulation efficiency, stability during storage and controlled release of the encapsulated carotenoid [133].

## 8. Novel Retinoid Pathway Therapeutic Targets

Several recent studies have suggested new therapeutic targets related to components of the retinoid pathway. For example, RORC, CRBP1, ALDH1A1 and TUBB3 (discussed in Chapter 3) have been proposed as potential therapeutic targets for BC. Preclinical in vitro experiments showed that increased expression of RORC suppressed cell proliferation and glucose metabolism and induced apoptosis in BC cells. Moreover, activation of RORC in BC cells increased cisplatin-induced apoptosis. These findings established RORC and RORC-mediated signalling as potential therapeutic targets for BC [51]. Increased expression of CRBP1 in transfected BC cell lines reduced cell growth and migration activity [50]. Moreover, in other cancers with decreased expression of CRBP1 (similar to BC), it was reported that forced overexpression of CRBP1 resulted in increased susceptibility to retinoids [134,135]. Namekawa et al. showed that ALDH1A1 and its putative downstream target TUBB3 could be exploited for therapeutic options in advanced disease [52]. Inhibition of ALDH1A1 by ALDH inhibitors and silenced ALDH1A1 expression by shRNA lentiviral transfer suppressed proliferation and spheroid formation of cancer cells from long-term BC patients. In addition, knockdown of TUBB3 also suppressed proliferation of these cells [52]. Taken together, these results suggest that RORC, CRBP1, ALDH1A1 and TUBB3 may be promising candidates for gene therapy or novel targets for improved adjuvant retinoid therapy of human BC.

Recently, a negative correlation between miR-29b, which functions as an oncogenic microRNA, and RARβ expression was demonstrated in a preclinical study of urothelial carcinoma [136]. The study showed that inhibition of miR-29b suppressed cell proliferation, growth, migration, invasion, and tumour growth via RARβ. Moreover, inhibitor of growth protein 4 (ING4) was identified as a tumour suppressor that directly interacts with RARβ. Silencing of ING4 reversed the RARβ-mediated suppression of cell migration and invasion. Thus, restoring RARβ and ING4 by inhibiting miR-29b may serve as a potential therapeutic target in BC [136].

## 9. Conclusions

Vitamin A and retinoids are already used in clinical practice for the treatment of some cancers. Many preclinical studies using various experimental models and designs have indicated that they could be used to decrease BC incidence and recurrence, as well as to improve BC therapy. However, the success of the few clinical trials conducted was limited due to inefficiencies, while the retinoid treatment was generally well tolerated. Although clinical trials are inconclusive, the accumulation of data on the limitations of the clinical use of retinoids has provided additional insight into the potential solutions. Currently, the most encouraging research is directed towards the development of novel complex platforms, such as synthetic retinoids without side effects, combinations of retinoids with chemotherapeutic agents and retinoid-related therapeutic targets that have already been tested for BC management. In addition, promising results are emerging with retinoids encapsulated into different types of delivery systems.

## Figures and Tables

**Table 1:** In vitro studies investigating the effects of retinoids in human BC cell lines.

Retinoids	In Vitro Model–Cell Line	Effects	Reference
ATRA ^1^	HT-1376	Inhibition of cell growth by inhibition of transcription factor AP-1 activity requiring RARα or RARβ mediated by the orphan receptor chicken ovalbumin upstream promoter-transcription factor (COUP-TF).	[85]
	RT112	Inhibition of epidermal growth factor (EGF)-induced cell growth.	[72]
	T24	Induction of apoptosis. Redistribution of apoptosis regulators Bax and Bcl-2, correlating with keratin 18 network reorganization.	[75]
		Induction of dose- and time-dependent cell proliferation. Downexpression of cellular retinol-binding protein-II (CRABP-II). Direct inhibition of peroxisome proliferator-activated receptor PPARβ/δ potentiating cell proliferation.	[86]
RA ^2^	EJ	Inhibition of cell growth and decreased expression of mutant p53.	[87]
4-HPR ^3^	T24	Increased expression of E-cadherin and translocation of β-catenin from the nucleus to the cytoplasm.	[78]
ATRA ^1^ 9-cis-RA ^4^ 13-cis-RA ^5^	RT4 T24	Inhibition of matrix metalloproteinases (MMPs).	[77]
ATRA ^1^ Bexarotene ^6^ 4-HPR ^3^ 9-cis-RA ^4^	RT4 T24 UM-UC-2/3/6/9/10/11/13/14	Resistance to ATRA and 9-cis-RA growth inhibition and apoptosis induction in most of the examined cell lines, which did not express RARβ. 4-HPR was the most potent growth inhibitor and apoptosis inducer.	[88]
ATRA ^1^ CD437 ^7^ 4-HPR ^3^	RT4 T24 UM-UC-2/3/6/10/13/14	Stronger effects on growth inhibition and apoptosis induction by synthetic retinoids (4-HPR and CD437) compared to natural (ATRA). Induction of expression of different nuclear retinoid receptors (RARα, RARβ, RARγ) by different retinoids.	[74]

^1^ ATRA, all-trans retinoic acid; ^2^ RA, retinoic acid; ^3^ 4-HPR, N-(4-Hydroxyphenyl)-retinamide or fenretinide; ^4^ 9-cis-RA, 9-cis-retinoic acid; ^5^ 13-cis-RA, 13-cis-retinoic acid; ^6^ bexarotene, also known as LGD1069 or Ro 26-445, brand name Targretin; ^7^ CD437, 6-[3-(1-adamantyl)-4-hydroxyphenyl]naphthalene-2-carboxylic acid, also known as AHPN or Ro 472077.

**Table 2:** In vivo studies investigating the effects of retinoids in carcinogen-based animal models of BC. In all experiments listed here, animals were fed with retinoid-supplemented diets.

Retinoid	In Vivo Model–Carcinogen (Species)	Effects	Reference
Bexarotene ^1^	BBN (rat)	Increased incidence and size of hyperplasia, papilloma and carcinoma.	[90]
Etretinate ^2^	BBN (rat)	Inhibition of urothelial papillary or nodular hyperplasia in a dose-dependent manner.	[92]
		No effect on BC.	[93]
Retinyl acetate	BBN (mouse)	Reduction of urothelial atypia and apoptosis in early BC.	[89]
	FANFT (mouse)	Inhibition of squamous and urothelial carcinomas.	[94]
4-HPR ^3^	BBN (mouse)	No reduction in tumour incidence.	[95]
	MNU (rat)	Inhibition of tumour growth when combined with the chemotherapeutic agent ADM.	[91]
13-cis-RA ^4^	BBN (rat)	Inhibition of urothelial carcinomas and other proliferative lesions of the bladder. Reduction in the incidence of hyperplasia, atypia, and urothelial carcinomas by simultaneous or delayed retinoid administration.	[96] [97]
	BBN (mouse)	Reduction in the incidence of invasive urothelial carcinoma in a dose-dependent manner.	[98,99]
	MNU (rat)	Inhibition of urothelial and squamous carcinomas and proliferative epithelial lesions by simultaneous or delayed retinoid administration.	[100,101]
ATRA ^5^ 13-cis-RA ^4^	MNU (rat)	Reduction in number and size of tumours.	[102]
ER ^6^ 2-HER ^7^ 13-cis-RA ^4^	BBN (rat, mouse)	Reduction in incidence, number, and severity of low-grade papillary urothelial carcinomas. ER and 2-HER were less toxic to rats than 13-cis-RA.	[103]
	FANFT (rat)	No inhibition of incidence or severity of BC.	[104,105]

^1^ Bexarotene, also known as LGD1069 or Ro 26-445, brand name Targretin; ^2^ etretinate, also known as ethyl etrinoate or Ro 10-9359, brand name Tigason; ^3^ 4-HPR, N-(4-Hydroxyphenyl)-retinamide or fenretinide; ^4^ 13-cis-RA, 13-cis-retinoic acid, ^5^ ATRA, all-trans retinoic acid; ^6^ ER, N-(ethyl)-all-trans-retinamide; ^7^ 2-HER, N-(2-hydroxyethyl)-all-trans-retinamide.

**Table 3:** Clinical trials investigating the chemopreventive effects of retinoids in BC.

Retinoid	BC Stage	Phase *Study Type*	No. of Retinod-Treated Patients (Mean Age)	No. of Control Patients (Mean Age)	Outcome	Reference
4-HPR ^1^	Ta, T1	Phase IIa	12 (68)	12 (65)	Well-tolerated side effects. Indication of reduced proliferation, delayed development of DNA aneuploidy or its reversal to diploidy.	[109]
		Phase IIb *randomized*	49 (63.8)	50 (61.6)	Well-tolerated side effects. No effect on DNA content distribution and morphology of urothelial cells. No effect on recurrence-free survival.	[110]
		*Twenty-year follow-up of randomized* [110]	33	29	No effect on outcome. Inverse association between baseline VEGF levels and BC survival.	[111]
		Phase IIb *randomized*	24 (60.1)	19 (61)	Lower IGF-I levels.	[112]
	Tis, Ta, T1	Phase III *randomized, placebo controlled*	70 (64.5)	67 (64.5)	Well-tolerated side effects. No effect on time-to-recurrence. Subgroup analysis indicated that high-risk patients co-treated with BCG had a lower risk of recurrence.	[113]
Etretinate ^2^	Ta, T1	Phase ND *randomized, placebo controlled*, *double-blinded*	15 (68.8)	15 (64.1)	Well-tolerated at final maintenance dose. Disturbing side effects at high doses. Preventive effect.	[114]
		Phase ND *Prospective randomized, placebo controlled, double-blinded*	37 (59.3)	42 (59.6)	Well-tolerated side effects. Cardiac toxicity in 3 patients. Similar first recurrence time but increased interval length for subsequent tumour recurrences.	[115]
	Recurring non-invasive bladder tumours	Phase ND *randomized, placebo controlled*	47	49	Patient dropout due to side effects (17 patients). No effect on outcome.	[116]
13-cis-RA ^3^	Ta, T1	Phase I/II	14	/	Toxicity and lack of positive results led to termination of the study.	[117]
**COMBINED TREATMENT**						
ATRA ^4^ + ketonazole	Ta, T1	Phase ND	16	25	Well-tolerated side effects. Improved survival time and decreased recurrence rate.	[118]
13-cis-RA ^3^ + entinostat	Epithelial tumours, including urothelial carcinoma	Phase I	18 (5 with BC)	/	Well tolerated. No objective responses were observed.	[119]

^1^ 4-HPR, N-(4-Hydroxyphenyl)-retinamide or fenretinide; ^2^ etretinate, also known as ethyl etrinoate or Ro 10–9359 (brand name Tigason); ^3^ 13-cis-RA, 13-cis-retinoic acid; ^4^ ATRA, all-trans retinoic acid; VEGF, vascular endothelial growth factor; ND, Not Defined.

## Abbreviations

4-HPR	N-(4-Hydroxyphenyl)-retinamide or fenretinide
ADH	alcohol dehydrogenase
ALDH	aldehyde dehydrogenase
ATRA	all-trans retinoic acid
BBN	N-butyl-N-(4-hydroxybutyl)-nitrosamine
BC	bladder cancer
BCG	Bacillus Calmette Guerin
BCO	β-carotene oxygenase
CD437	6-[3-(1-Adamantyl)-4-hydroxyphenyl]-2-naphthalene carboxylic acid
CIS	carcinoma in situ
CRABP	cellular retinoic acid-binding protein
CRBP	cellular retinol-binding protein
CSC	cancer stem cell
CYP26	cytochrome P450 26
EGF	epidermal growth factor
FABP5	fatty acid-binding protein 5
FANFT	N-4-(5-nitro-2-furyl)-2-thiazolylformamide
LRAT	lecithin retinol acyltransferase
MIBC	muscle invasive bladder cancer
MMPs	matrix metalloproteinases
NMIBC	non-muscle invasive bladder cancer
NMU	N-methyl-N-nitrosourea
PPAR	peroxisome proliferator-activated receptor
PUNLMP	papillary urothelial neoplasm of low malignant potential
RA	retinoic acid
RALDH	retinal dehydrogenase
RAR	retinoic acid receptor
RARE	retinoic acid response element
RBP4	retinol-binding protein 4
RDH	retinol dehydrogenase
RORC	retinoic acid–related orphan receptor C
RXR	retinoid X receptor
STRA6	stimulated by retinoic acid 6
TNM	Tumour, Node, Metastasis
VAD	vitamin A deficiency
